# SARS-CoV-2 nonstructural protein 6 from Alpha to Omicron: evolution of a transmembrane protein

**DOI:** 10.1128/mbio.00688-23

**Published:** 2023-07-21

**Authors:** Shuchen Feng, Amornrat O'Brien, Da-Yuan Chen, Mohsan Saeed, Susan C. Baker

**Affiliations:** 1 Department of Microbiology and Immunology, Stritch School of Medicine, Loyola University Chicago, Chicago, Illinois, USA; 2 Department of Biochemistry and Cell Biology, Boston University Chobanian and Avedisian School of Medicine, Boston, Massachusetts, USA; 3 National Emerging Infectious Diseases Laboratories (NEIDL), Boston, Massachusetts, USA; University of Hong Kong, Pokfulam, Hong Kong

**Keywords:** SARS-CoV-2, nonstructural protein 6, nsp6, SARS-CoV-2 evolution, nsp6 topology model, SARS-CoV-2 attenuation

## Abstract

We recently reported that mutations in both the spike glycoprotein and nonstructural protein 6 (nsp6) were associated with attenuation of the SARS-CoV-2 Omicron BA.1 variant. While mutations in spike allow evasion of neutralizing antibodies and promote specific modes of viral entry, the role of nsp6 mutations in pathogenesis is less clear. Nsp6 is essential for modifying the endoplasmic reticulum and generating double-membrane vesicles, the site of viral RNA replication. To investigate the evolution of nsp6, we evaluated 91,596 high-confidence human SARS-CoV-2 whole-genome sequences across 19 variants and lineages. While nsp6 of early variants of concern, such as Alpha, Beta, and Gamma, carried a triple amino acid deletion (106–108, termed ΔSGF), the Delta, Epsilon, and Mu lineages retained the ancestral nsp6 sequence. For nsp6 in the emerging Omicron variants, we report a transition from an amino acid 105–107 ΔLSG deletion in BA.1 to increased dominance of the ΔSGF in BA.2 and subsequent lineages. Our findings indicate that deletion within nsp6 was independently selected in multiple lineages of SARS-CoV-2, both early and late in the pandemic. Analysis of SARS-CoV-2-related coronaviruses in bats and pangolins revealed nsp6 sequences similar to the ancestral SARS-CoV-2 virus, indicating that the deletion in nsp6 may be an adaptation to replication in humans. Analysis of nsp6 sequences from multiple coronaviruses predicts a multipass transmembrane protein with a conserved C-terminal domain. Monitoring and evaluating changes in nsp6 and other nonstructural proteins will contribute to our understanding of factors associated with the attenuation of pandemic coronaviruses.

**IMPORTANCE:**

There is an ongoing need to evaluate genetic changes in SARS-CoV-2 for effects on transmission and pathogenesis. We recently reported an unexpected role for replicase component nsp6, in addition to changes in spike, in the attenuation of Omicron BA.1. In this commentary, we document a triple-amino-acid deletion in a predicted lumenal domain of nsp6 that was found in multiple, independent variants of SARS-CoV-2, including all recent Omicron lineages. Furthermore, we modeled the predicted structure of nsp6, implicating a multipass transmembrane architecture as conserved in members of the *Coronaviridae* family. This information can guide future studies investigating the role of nsp6 in the pathogenesis of existing and emerging coronaviruses.

## OBSERVATION

One major challenge of the ongoing COVID-19 pandemic is that emerging variants of SARS-CoV-2 may escape neutralizing antibodies generated by vaccination and/or previous infection. To date, variants of concern (VOCs) designated Alpha to Omicron have been documented (1). Substitutions and deletions in the spike (S) glycoprotein facilitate escape from neutralizing antibodies and can result in alternative mechanisms of virus entry (2, 3). Additionally, changes in the nucleocapsid protein have been associated with increased virulence, likely due to enhanced packaging of viral RNA (4).

Since its emergence in November 2021 in South Africa (5), the Omicron variant has been associated with increased transmission but attenuated virulence compared to the ancestral virus (Wuhan-Hu-1) or the Delta VOC in multiple studies of animal models (6, 7) and human outcomes (8, 9). Prior vaccination or infection may also mitigate the pathogenesis of emerging variants (10). However, the contributing mutations were unknown. Recently, we reported an unexpected finding that mutations in spike and nonstructural protein 6 (nsp6), one of the 16 non-structural proteins generated by the proteolytic processing of the viral pp1ab polyprotein, were associated with the attenuation of Omicron BA.1 in K18-hACE2 mice (11). We found that the Omicron spike alone was not sufficient to fully attenuate the virulence of SARS-CoV-2, yet the combination of both Omicron spike and Omicron nsp6 attenuated the virulence to the level of Omicron BA.1. These findings were corroborated by a recent study that employed chimeric combinations of Delta and Omicron variants, identifying the predominant role of spike and nsp6 in Omicron BA.1’s attenuation (12). Omicron BA.1 nsp6 contains a triple amino acid deletion (105–107, ΔLSG) and an amino acid substitution (I189V). Here, we provide an updated analysis on the evolution and predicted structure and function of SARS-CoV-2 nsp6.

### Nsp6 deletion mutants found in early variants enhance ER membrane zippering

**Fig 1:**
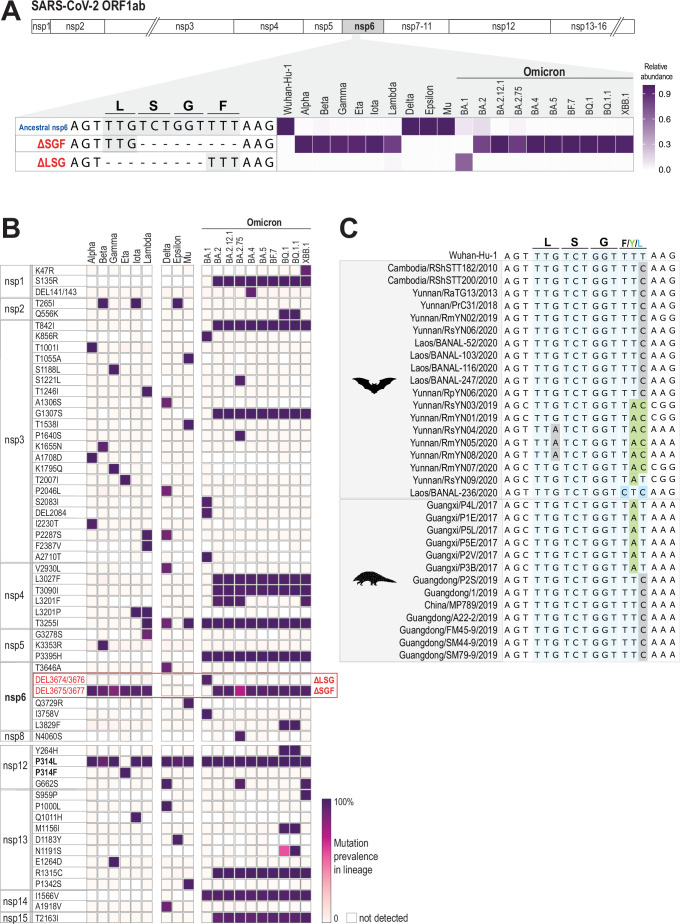
Analysis of SARS-CoV-2 nsp6 reveals SGF/LSG deletions in 16 of 19 lineages. (**A**) Relative abundance of deletion of SGF and LSG sequences in the ancestral SARS-CoV-2 strain (Wuhan-Hu-1) and 19 subsequent lineages. The evaluated human SARS-CoV-2 whole-genome sequences are downloaded from the Global Initiative on Sharing Avian Influenza Data (GISAID) platform with a limit to a maximum of 5,000 sequences per variant except for Alpha and Delta, where more sequences are included. Sequences are screened to be “high coverage” or “low coverage excluded,” “collection date complete,” and include those submitted from sites around the world. The prevalence of the mutation types in each variant/lineage is shown on the right, with colors from light blue to deep pink representing increased values. (**B**) Analysis using Outbreak.info documents the prevalence of mutations in the ORF1ab region across the 19 lineages (accessed on 6 January 2023). The nsp6 deletion region is highlighted in the red box. The mutation prevalence in each variant/lineage is indicated by the intensity of the purple color. (**C**) Analysis of the nucleotide sequence encoding LSGF in nsp6 from viruses isolated from 19 bats and 13 pangolins. The alignment of the LSGF region is shown in light blue. Synonymous mutations are highlighted in gray and non-synonymous in green (F to Y) and blue (F to L).

Previous studies documented a critical role of nsp6 in coronavirus replication via the formation of an nsp3-nsp4-nsp6 complex, a pre-requisite for the biogenesis of double-membrane vesicles (DMVs) (13 - 16). DMVs are the site of viral RNA synthesis for coronaviruses (17, 18). SARS-CoV-2, like other coronaviruses, induces ER-derived DMVs as part of the viral replication and transcription mechanisms (18 - 20). An elegant study by Ricciardi and co-workers suggested that SARS-CoV-2 nsp6 is responsible for organizing and connecting the DMVs to the ER in a process termed ER zippering (21). Interestingly, they reported that nsp6 with a triple amino acid deletion (106–108, ΔSGF) found in early VOCs (Alpha, Beta, and Gamma) conferred an increased ER-zippering activity. This ΔSGF deletion partially overlaps with the 105–107 ΔLSG deletion in Omicron BA.1 nsp6 (Fig. 1A). We also noted that the ancestral sequence of nsp6 is maintained in the more pathogenic Delta VOC. These observations of different nsp6 sequences in VOCs motivated us to evaluate the evolution of nsp6 over the course of the pandemic.

### Amino acid deletions in nsp6 are independently selected for human adaptation

To understand SARS-CoV-2 nsp6’s evolution, we examined 91,596 human SARS-CoV-2 whole-genome sequences across 19 variants and/or lineages obtained from the Global Initiative on Sharing Avian Influenza Data (GISAID) platform (Table S1). The nsp6 ΔSGF deletion is found in early variants, including Alpha, Beta, Gamma, and later Eta, Iota, and Lambda (21), reported in 95% of the sequences. In contrast, the triple amino acid deletion is not present in Delta, Epsilon, and Mu VOCs, where the ancestral nsp6 sequence is retained in 99.6% of these variants. The Omicron BA.1 lineage has the most diversity in the reported sequences, with 65% ΔLSG, 8.5% ancestral nsp6, 0.1% ΔSGF, and a surprisingly high number of sequences that would result in a frameshift in nsp6 (10.3% 5-nucleotide deletion; 4.3% 2-nucleotide deletion; 11.7% other nsp6 nucleotide changes, *n* = 4,976). The 5- and 2-nucleotide deletions (~15% of BA.1 sequences) could be sequencing or assembly errors in the consensus genomes available on GISAID. Currently, it is unclear why these deletions are more prevalent in BA.1 genomes compared to other Omicron sublineages. Interestingly, the ΔSGF nsp6 again becomes the dominant deletion type in all Omicron VOCs from BA.2 to XBB. This deletion is reported in 78% of BA.2, 83% in BA.2.75 sequences, and >97% in the more recent Omicron lineages. The accession numbers for the 91,596 genomes evaluated for this study are available upon request.

When extending our scope to evaluate all mutations in the ORF1ab region, we identified only one additional mutation retained consistently across all variants, ORF1b:P314L in nsp12 (Fig. 1B). This mutation was reported as a co-evolution with the spike D614G substitution (22, 23). Figure 1B also shows that Omicron variants encode multiple mutations in ORF1ab compared to earlier lineages. The relative contribution of individual or combined mutations in ORF1ab to the attenuation of Omicron variants remains to be determined.

Taken together, the dominance of the nsp6 ΔSGF/ΔLSG deletion in multiple, independent VOCs (Fig. 1; Table S1) suggests that SARS-CoV-2 nsp6 ΔSGF/ΔLSG deletions may have been selected for viral adaptation in humans.

### Nsp6 deletion mutants are not present in bat or pangolin coronaviruses

To determine if the ΔSGF/ΔLSG deletions were present in nsp6 of other SARS-like viruses, we compared whole-genome sequences of bats and pangolins coronaviruses to the SARS-CoV-2 reference genome (NCBI GenBank accession number NC_045512.2), including the bat coronaviruses that share the highest nucleotide identity to human SARS-CoV-2 (Yunnan/RaTG13/2013 and Laos/BANAL/2020) (24 - 27) (Fig. 1C). We show that these bat and pangolin coronaviruses do not carry any deletion in this region of nsp6; actually, they are quite similar to the ancestral SARS-CoV-2, with the same codons for the 106S and 107G amino acids. Synonymous mutations at the 108^th^ position (F) are observed (indicated in gray color, Fig. 1C), resulting in 11 of bat (58%) and 7 of pangolin (54%) CoV genomes with identical amino acids to that of ancestral SARS-CoV-2 nsp6. This consistency of nsp6 in bat and pangolin coronaviruses and SARS-CoV-2 further supports that the nsp6 deletion found in VOCs may represent an adaptation of SARS-CoV-2 to the human host.

### Predicting the topology of SARS-CoV-2 nsp6

**Fig 2:**
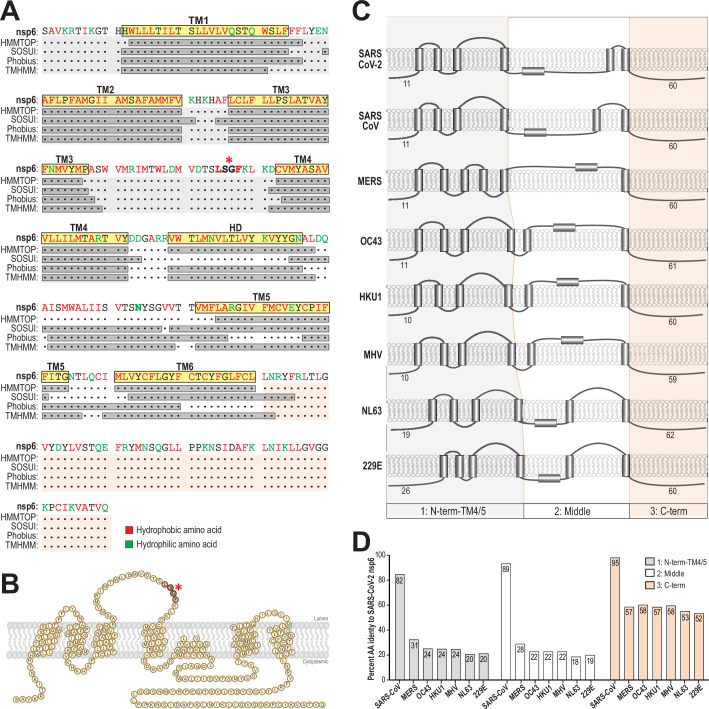
Analysis of predicted hydrophobic and transmembrane domains of nsp6 from seven human coronaviruses and mouse hepatitis virus (MHV). (**A**) Sequences of SARS-CoV-2 nsp6 (NCBI accession number YP_009725302.1) are analyzed for the prediction of the hydrophobic domain (HD) and transmembrane domain (TM) using HMMTOP, SOSUI, Phobius, and TMHMM programs shown in gray boxes. The residues predicted as likely HD or TM by three of the four programs are indicated in the yellow boxed areas. Red and green fonts represent hydrophobic and hydrophilic amino acids, respectively. (**B**) The diagram illustrates a proposed model of the topology of SARS-CoV-2 nsp6. The asterisk indicates the position of LSGF residues located in between TM3 and TM4. (**C**) Alignment of predicted topologies for six beta coronaviruses (SARS-CoV-2, SARS-CoV [NP_828864.1], MERS-CoV [YP_009047218.1], HCoV-OC43 [YP_009924324.1], HCoV-HKU1 [YP_009944274.1], and MHV-A59 [YP_009915678.1]) and two alpha coronaviruses (HCoV-NL63 [YP_010229075.1] and HCoV-229E [NP_073549.1]). The topologies were predicted by three of the four programs as described previously. (**D**) Amino acid identity in the N-terminal, middle, and C-terminal domains of nsp6 reveals more conservation in the C-terminal domain.

The deletion mutations in nsp6 and its association with the attenuated Omicron phenotype raise several questions. Exactly where is this deletion in the overall structure of nsp6 and what function(s) are altered in the mutant? To date, there is no structural information available for SARS-CoV-2 nsp6. Previous studies using the model murine coronavirus, mouse hepatitis virus (MHV), revealed that nsp6 had both its termini exposed on the cytoplasmic face of the ER membrane while spanning the lipid bilayer six times (14, 28). Here, we analyzed the structure of nsp6 in SARS-CoV-2 with comparison to other human coronaviruses using multiple programs: HMMTOP, SOSUI, Phobius, and TMHMM (29 - 32) (Fig. 2A and C). Our analysis agreed with the six transmembrane (TM) topologies and identified a likely hydrophobic domain (HD) as membrane-associated in SARS-CoV-2 nsp6. All programs revealed similar helical structures for the first four and the final TM domains. The deletion mutation (ΔSGF/ΔLSG) found in VOCs is located within the predicted second and longest luminal loop (Fig. 2B). Further comparison of SARS-CoV-2 nsp6 with six other human coronaviruses and MHV suggested that the number of predicted TM domains and the likely-HD is highly conserved (Fig. 2C). Interestingly, the non-SARS viruses had an overall high amino acid identity and conserved sequence length in the C-terminal domain, while the N-terminal and middle domains showed lower identity in amino acid sequences (Fig. 2D), suggesting that the C-terminal domain may play an important role in nsp6 function.

Targeting nsp6 may be productive for limiting the replication of potentially emerging coronaviruses. Previous studies point to the essential role of nsp6 in the assembly and ER tethering of viral replication complexes and in the recruitment of lipid droplets to DMVs, whose association is thought to be essential for the maintenance and proliferation of viral replication organelles (14, 16, 21). Further studies are needed to identify host factors interacting with nsp6 during membrane zippering and DMV formation as well as to elucidate the role of the protein C-terminal domain and luminal and cytoplasmic loops in viral replication.

## SUPPLEMENTAL MATERIAL

Table S1 mbio.00688-23-s0001.eps Sequence types of the nsp6 deletion region across 19 SARS-CoV-2 variants and/or lineages.

ASM does not own the copyrights to Supplemental Material that may be linked to, or accessed through, an article. The authors have granted ASM a non-exclusive, world-wide license to publish the Supplemental Material files. Please contact the corresponding author directly for reuse.
